# A Systematic Review of Smoking Cessation Interventions for Adults in Substance Abuse Treatment or Recovery

**DOI:** 10.1093/ntr/ntv127

**Published:** 2015-06-11

**Authors:** Sarah L. Thurgood, Ann McNeill, David Clark-Carter, Leonie S. Brose

**Affiliations:** 1 ^1^ Centre for Health Psychology, Staffordshire University, Stoke-on-Trent, United Kingdom;; 2 ^2^ Department of Addictions, UK Centre for Tobacco and Alcohol Studies (UKCTAS), Institute of Psychiatry, Psychology and Neuroscience, King’s College London, London, United Kingdom

## Abstract

**Introduction:**

The aim was to evaluate the effectiveness of smoking cessation interventions for patients with substance use disorders. The secondary aim was to evaluate impact on substance use treatment outcomes.

**Methods:**

Randomized controlled trials involving adult smokers, recently or currently receiving inpatient or outpatient treatment for substance use disorders were reviewed. Databases, grey literature, reference lists, and journals were searched for relevant studies between 1990 and August 2014. Two authors extracted data and assessed quality. The primary outcome was biochemically verified continuous abstinence from smoking at 6 or 12 months, secondary outcomes were biochemically verified 7-day point prevalence smoking abstinence (PPA) at 6 or 12 months and substance use outcomes. Heterogeneity between studies precluded pooled analyses of the data.

**Results:**

Seventeen of 847 publications were included. Five studies reported significant effects on smoking cessation: (1) nicotine patches improved continuous abstinence at 6 months; (2) nicotine gum improved continuous abstinence at 12 months; (3) counseling, contingency management and relapse prevention improved continuous abstinence at 6 and 12 months; (4) cognitive behavioral therapy, plus nicotine replacement therapy (NRT), improved PPA at 6 months; and (5) a combination of bupropion, NRT, counseling and contingency management improved PPA at 6 months. Two studies showed some evidence of improved substance use outcomes with the remaining eight studies measuring substance use outcomes showing no difference.

**Conclusions:**

NRT, behavioral support, and combination approaches appear to increase smoking abstinence in those treated for substance use disorders. Higher quality studies are required to strengthen the evidence base.

## Introduction

Tobacco use is one of the main risk factors for many chronic illnesses including cancer, lung diseases, and cardiovascular diseases, making it one of the largest preventable causes of premature death and disease across the world. Tobacco kills nearly 6 million people each year and this could rise to 8 million by 2030.^1^

Smoking is particularly prevalent in the population of those in treatment or recovery from substance use disorders. Between 74% and 98% of patients with substance use disorders are also smokers.^2–6^ Many patients with substance use disorders may overcome their primary addiction and then die from a tobacco related illness.^7^ For example, in a cohort of former alcoholics and patients with drug dependence, 51% of deaths were from tobacco related causes.^8^

Smoking may be seen as beneficial when giving up alcohol or drugs,^9^ for example, through perceptions that it is calming or lessens anxiety, but such perceptions are likely due to misattribution given smoking relieves nicotine withdrawal.^10^ In general, smoking has been shown to be an ineffective coping strategy and may worsen substance use treatment outcomes.^11,12^

The negative health effects of smoking and the benefits of offering smoking cessation to patients with substance use disorders are well known but more research is needed to identify successful interventions.^5,13^ Medication such as nicotine replacement therapy (NRT), varenicline, bupropion, cytosine, and behavioral support increases success of quit attempts in the general population of smokers, particularly when combined^14–18^ and have been recommended as effective interventions within clinical guidelines for the general population.^19^ Clinical guidelines for substance use services^20^ state that “Secondary care providers have a duty of care to protect the health of, and promote healthy behaviour among, people who use, their services. This duty of care includes providing effective support to stop smoking or abstain from smoking while using secondary care services” (pp.5–6). Smokers should be identified at the first opportunity and provided with advice and support.^19^ This includes providing pharmacotherapy to support abstinence along with an offer of arranging intensive behavioral support. Common assumptions include that the level of motivation to quit within patients with substance use disorders is low; however, it has been found that patients receiving treatment for substance use disorders are as motivated to quit as smokers in the general population.^6,21,22^ A further concern is that tobacco cessation will compromise substance use treatment, however a previous review indicates that smoking cessation improves abstinence from alcohol and illicit drugs.^11^

This review aimed to evaluate two main questions: (1) what is the effectiveness of different smoking cessation interventions for patients with substance use disorders? and (2) what is the impact of smoking cessation treatment on substance use outcomes? This extends and updates the findings of a previous meta-analysis.^11^ The quality of included studies was also evaluated. As many substance use disorder services do not offer smoking cessation treatment,^23^ it is hoped that the findings will provide a clearer direction of how to incorporate smoking cessation into substance use treatment.

## Methods

### Search Strategy

Searches were conducted by SLT in February 2014 and updated in August 2014. Included literature was published from 1990 onwards. Databases searched were Cinahl, Medline, Psycharticles, Psychbooks, Centre for Reviews and Dissertations, British Library, Web of Science, Science Direct, The Cochrane Library, and Swetswise. The search strategy included searching for grey literature using Open Grey, Grey Net, The Medical Research Council, Ethos (British Library), The Universal Index of Doctoral Dissertations, and The Conference Proceedings Citation Index. Words relating to smoking cessation and substance use disorders, using combinations of “or” and “and” or free text were adapted as required for each database. The thesaurus and MeSH terms were used to identify associated search terms. The final list of search terms is provided as a Supplementary Material S1.

To identify any additional studies of relevance to the research questions, a search was conducted in the Journal of Substance Abuse Treatment, Drug and Alcohol Dependence, Addictive Behaviours, and Addiction for editions between January 2010 and January 2014 as these journals had published the majority of the papers identified for review. Manual searches of reference lists and citation searches were also completed.

### Inclusion and Exclusion Criteria

To review the most methodologically sound studies, only randomized controlled trials of smoking cessation interventions with at least 6 months follow-up, involving smokers over the age of 18, who had recently completed or were currently receiving inpatient or outpatient treatment for a substance use disorder (drugs or alcohol) were included. Interventions could include pharmacological and nonpharmacological approaches in any setting and mode of delivery. Trials had to include two different treatments, one of which could be placebo or usual care. Trials comparing different timings such as concurrent versus delayed implementation of the same intervention were therefore excluded. The question of timing is the subject of a proposed Cochrane review.^24^ In addition, studies had to report biochemically-verified smoking abstinence outcomes in accordance with the Russell Standard.^25^

### Outcome Measures

The primary outcome measure was biochemically verified (carbon monoxide) self-reported continuous abstinence from smoking, at the 6- or 12-month follow-up.^25,26^ Secondary outcome measures were biochemically verified self-reported 7-day point prevalence abstinence from smoking (PPA) at 6- or 12-month follow-up.^25,27^ Substance use treatment outcomes at 6 or 12 months were also included. Different studies reported different substance use treatment outcomes. Three studies defined substance use abstinence as self-reported, no substance use over the last week confirmed by urine drug screen or breath alcohol test results.^28–30^ Two studies defined 30-day point prevalence substance use abstinence as self-reported, no substance use during the 30 days prior to follow-up combined with a breath alcohol test^31^ or a breath alcohol and a urine drug screen.^32^ One study defined continuous substance use abstinence as self-reported, no substance use over the 90 days prior to the follow-up time point confirmed by urine drug screen or breath alcohol test results.^33^ One study defined the proportion of heavy drinking days as the amount of heavy drinking days, 14 days prior follow-up and the 30 days prior to the 3- and 6-month follow-up. A heavy drinking day was defined as any day on which a man drank six or more standard drinks, or a woman drank four or more standard drinks.^34^ Because little evidence on the effectiveness of interventions in this population is available, for studies reporting no effect at 6 or 12 months, earlier follow-up comparisons were included as secondary outcomes.

### Data Analysis

SLT screened the initial titles. SLT and LSB screened the abstracts and full papers for relevance to the research question. Reasons for exclusion were documented and any discrepancies discussed amongst the reviewers to arrive at a consensus (excluded references in Supplementary Table S2).

Data extraction was carried out by SLT and LSB using a data extraction sheet designed for randomized controlled trials.^35^ The data extraction process recorded the following: study aims, population, eligibility criteria, randomization methods, sample characteristics, intervention type, study setting, recruitment method, study staff, substance type, smoking and substance use outcomes measures, follow-up period, and study findings. There was a 95% agreement rate between reviewers and disagreements were resolved through discussion and rechecking the papers. Quality assessment was carried out by SLT and LSB using the Cochrane tool for assessing bias.^35^ Where statistical information was available from the paper or by contacting the author, findings were computed into a common effect size statistic of *r*.^36^

Due to heterogeneity in terms of the population, control group, length of follow-up and outcome measures, there were no sets of studies that were sufficiently similar to make them suitable for inclusion in a meta-analysis, so a narrative synthesis was used.

## Results

**Figure 1.**
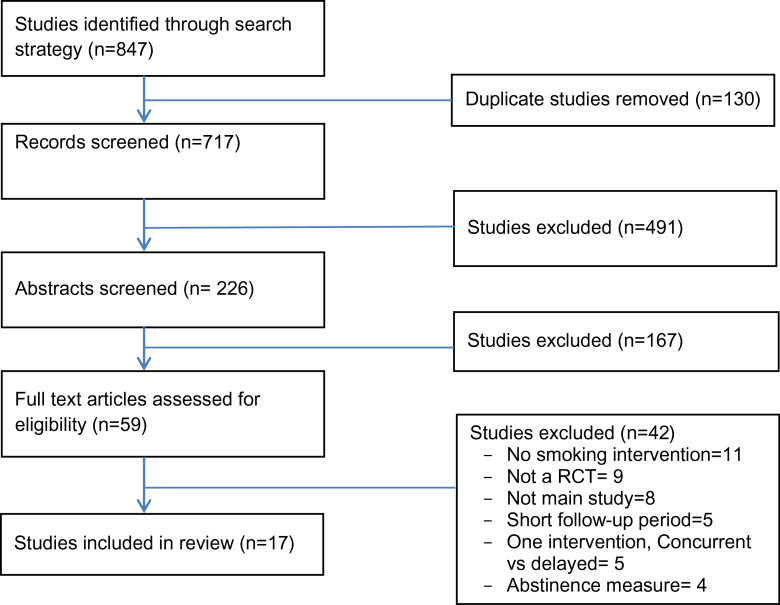
Search process and study selection.

The screening process led to the identification of 17 studies for inclusion in the review. Figure 1 contains a flow diagram^37^ of the screening process. Study characteristics are summarized in Table 1. Study sample size ranged from 64 to 383 participants. Mean age of the participants ranged from 34 years to 50 years. One study contained 100% males, otherwise the percentage of males ranged from 50% to 97%. Mean baseline number of cigarettes smoked per day ranged from 16 to 32.

**Table 1.** Study Characteristics

Author	Outcome measures	Follow-up (months)	Setting	Population	Sample size	Substance type	Mean baseline cigarettes per day (*SD*)	Mean age (*SD*)	Percentage male
Burling et al.^32 ^	7-day point prevalence CO level	1,3,6, and 12	Inpatient residential substance abuse treatment	Newly recovering drug and alcohol dependent cigarette smokers (30 days abstinent)	100	Alcohol, cocaine, or both	18 (7.4)	40 (6.2)	95%
Carmody et al.^38 ^	7-day point-prevalence verified by CO < 10 ppm, continuous abstinence	3,6.5,9.5,12	Outpatient substance abuse clinic.	Alcohol-dependent smokers in early recovery (at least 7 days abstinent)	162	Alcohol	17 (10.2)	50 (nr)	97%
Cooney et al.^34 ^	7-day point-prevalence verified by CO < 10 ppm, time to relapse	1, 3.5, and 6.5	Outpatient substance abuse clinic	Smokers who had alcohol dependence in the last 3 months (excluded if any current drug use)	118	Alcohol	25 (9.9)	47 (7.8)	89%
Cooney et al.^33 ^	Continuous abstinence, time to smoking relapse.	3,6, and 12	Outpatient substance abuse clinics	Smokers in early treatment for current alcohol abuse or dependence	96	Alcohol	25 (10.3)	45 (10.1)	71%
Gariti et al.^28 ^	7-day point-prevalence verified by CO < 9 ppm	1, 6, and 12	Inpatient residential substance abuse treatment	Smokers with current substance dependence.	64	Alcohol, cocaine, opiates	24 (nr)	44 (nr)	100%
Hays et al.^39 ^	7-day point-prevalence verified by CO < 8 ppm	12 and 17	Outpatient community sample	Smokers with a past history of alcohol abuse or dependence and had at least 1 year of continuous abstinence from alcohol and drugs.	195	Alcohol or drugs	30 (11.8)	44 (9.4)	77%
Hughes et al.^40 ^	Continuous abstinence plus CO <10 ppm	6.5	Outpatient alcohol treatment	Smokers with a past history of alcohol dependence with no substance misuse in last 30 days.	115	Alcohol	30 (11)	43(9)	72%
Kalman et al.^41 ^	7-day point-prevalence verified by CO < 10 ppm	1,3,6, and 9	Outpatient community based substance abuse treatment	Smokers with a past history of alcohol dependence and at least 2 months of abstinence from any substances.	130	Alcohol	32 (10.2)	47 (8.2)	84%
Kalman et al.^42 ^	7-day point-prevalence verified by CO < 8 ppm, continuous abstinence	6	Inpatient substance abuse treatment	Smokers with a history of alcohol or dependence and between 2 and 12 months of abstinence.	148	Alcohol	21 (11.2)	42 (9.6)	83%
Mueller et al.^29 ^	7-day point-prevalence verified by CO < 10 ppm and urine cotinine level	6	21-day inpatient alcohol detoxification unit	Alcohol dependent smokers currently in alcohol detoxification treatment	103	Alcohol	26 (12.1)	44 (11)	84%
Patten et al.^43 ^	7-day point-prevalence verified by CO < 10 ppm	6 and 12	Outpatient community Sample	Smokers with a past history of alcoholism (an average of 4 years abstinence from alcohol and drugs).	205	Alcohol	28 (11.9)	42 (9.9)	55%
Reid et al.^30 ^	7-day point-prevalence verified by CO < 10 ppm	3 and 6.5	Outpatient community- based treatment programs	Smokers with current drug or alcohol dependence enrolled in treatment for at least 30 days.	225	Alcohol or drugs	22 (11.6)	42 (10.2)	51%
Rohsenow et al.^44 ^	7-day point-prevalence verified by CO < 10 ppm	1,3,6, and 12	Inpatient residential substance abuse treatment	Smokers with current alcohol dependence.	165	Alcohol and drugs	21 (8)	34 (7.6)	60%
Shoptaw et al.^45 ^	7-day point prevalence confirmed by CO < 8 ppm	6 and 12	Outpatient narcotic treatment clinic	Current methadone maintained smokers	175	Methadone treatment	23 (13.2)	44 (7.3)	68%
Stein et al.^46 ^	7-day point-prevalence verified by CO < 8 ppm	1,3, and 6	Outpatient methadone treatment	Methadone maintained smokers	383	Methadone treatment	27 (12.2)	40 (8.4)	53%
Stein et al.^47 ^	7-day point-prevalence verified by CO < 8 ppm	6	Outpatient substance abuse treatment clinic	Methadone maintained smokers	315	Methadone treatment	20 (10.4)	40 (9.7)	50%
Winhusen et al.^48 ^	7-day point-prevalence verified by CO < 8 ppm	3 and 6	Outpatient substance use treatment clinic	Current stimulant dependent smokers	267	Cocaine methamphetamine	16 (7.9)	37 (10)	89%

CO = carbon monoxide; nr = figures not reported within the article.

Eight studies investigated smoking cessation in alcohol treatment only, five investigated alcohol and drug treatment and four investigated drug treatment only. Studies included one or more of the following addictive substances: alcohol, cocaine, heroin, cannabis, amphetamines, methamphetamines, and benzodiazepines.

### Treatment Status

Five studies investigated smokers currently in treatment for a substance use disorder. Four studies stated that patients were currently in treatment but enrolled in the intervention after a specific period of abstinence (eg, 7 days abstinence^38^, after 30 days abstinence^30,32^ or had a substance use diagnosis in the past 3 months^49^). Three studies investigated methadone-maintained patients. Five of the studies investigated smokers with a past history or drug or alcohol abuse. This tended to relate to a specific period of abstinence (eg, at least 2 months abstinence,^41^ between 2 and 12 months abstinence^42^ or at least 1 year of abstinence^39^). One study stated that participants had an average of 4 years abstinence^43^ and another study participants had a median of 5 years of abstinence.^40^

### Interventions

#### Setting

Twelve studies were conducted in outpatient and five in inpatient settings. Intervention settings included long-term and short-term residential treatment, outpatient community treatment centers, and outpatient methadone clinics.

#### Recruitment Method

Six studies reported recruitment methods, including advertisements in local newspapers or on clinic bulletin boards and asking people to contact the research team to take part. Other methods included recruiting patients through nominations from treatment staff. Three studies used Alcoholics Anonymous (AA) meetings as a way to recruit patients, including one study which appointed members of the AA group as study recruiters. One study gave presentations at treatment clinics until enough patients had enrolled. Overall, 12 studies either required participants to have an intention to quit smoking or recruited participants via posters or other advertising, suggesting that participants were likely to have had some intention or motivation to quit.

#### Smoking Outcome Measures

One study reported only continuous abstinence at 6 months, another at 6- and 12-month follow-up. Four studies reported continuous abstinence and PPA: two at 6 months, one at 6 and 12 months and one at 12 months. Eleven studies only measured PPA, as the primary smoking outcome; of these, seven studies reported smoking outcomes at 6 months only, three studies reported smoking outcomes at 6 and 12 months and one reported smoking outcomes at 12 months.

#### Intervention Characteristics

The intervention characteristics can be seen in Supplementary Table S3. The studies used a variety of different staff members to deliver the interventions, including research staff, therapists, nurses, physicians, and psychiatrists. The main intervention categories included counseling only; counseling and NRT; NRT only; cognitive behavioral treatment (CBT) only; CBT and NRT; motivational interviewing; bupropion and varenicline. One approach, used in three of the interventions^32,45,48^ was contingency management (CM) which involves positive reinforcement (use of financial or material incentive) to promote desired behaviors.^50^

### Effectiveness of Smoking Cessation Interventions

Five of the 17 studies reported significant effects at 6- or 12-month follow-up. One study^40^ found significant differences in continuous abstinence at 6 months, another study^33^ found significant differences in continuous abstinence at 12 but not at 6 months and a further study found significant differences in continuous abstinence at both 6 and 12 months.^32^ Two studies reporting PPA as primary outcome^31,48^ found significant differences between interventions, both at 6 months. Four of the studies provided smoking cessation treatment concurrently with substance use treatment and one provided treatment for patients with a past history of a substance use disorder. Results are detailed in Supplementary Table S3.

Two studies found evidence that NRT is of benefit.^33,40^ For outpatient smokers with a past history of alcohol dependence, 21-mg nicotine patches significantly increased continuous abstinence at 4 months and at 6 months compared with a placebo patch.^40^ A combination of CBT, nicotine patch and nicotine gum had significantly higher continuous abstinence rates at 12 months than the CBT, nicotine patch and placebo gum condition^33^ for outpatient smokers in early treatment for alcohol dependence.

One study found evidence that a behavioral support is of benefit.^32^ A multicomponent smoking treatment (MST) consisting of 5 weeks of prequit treatment, 4 weeks of postquit counseling, individual daily counseling sessions, contingency contracting, and relapse prevention training was compared against the same MST plus generalization training (MST+G), and a usual care condition^32^ for inpatient, newly recovering drug and alcohol dependent smokers. All participants received nicotine patches. The relapse prevention training identified high risk situations and practiced coping skills that could be used in these situations. The generalization training similarly identified high risk situations common to smoking, drug and alcohol use, and involved practicing coping skills to prevent relapse to all substances. Continuous abstinence differed significantly between groups both at 6 and 12 months, with both intervention groups achieving higher rates than the usual care group. There were no significant differences in PPA at the 6-month or at 12-month follow-up; although the two intervention groups combined and separately had higher than the usual care group at 1-month postquit.

Two studies found evidence that a combination of behavioral support and medication was beneficial to smoking cessation.^31,48^ Carmody^31^ provided an intensive intervention consisting of 16 sessions of individualized CBT, 16 weeks of nicotine patches and 26 weeks of nicotine lozenges in comparison with usual care which included a referral to a smoking cessation clinic. PPA was significantly higher for the intensive intervention group at 12 weeks for outpatient alcohol dependent smokers in early recovery and at 6 months but there was no significant difference at 12 months. Winhusen^48^ randomized outpatient, current stimulant dependent smokers into substance use treatment as usual or substance use treatment as usual plus smoking cessation treatment. Smoking cessation treatment included weekly individualized counseling and bupropion. Further into the intervention, participants were also offered a nicotine inhaler and CM. PPA was significantly higher in the intervention group compared to the treatment as usual group at 10 weeks, 3 months, and at 6-month follow-up.

Of the 12 studies that did not find treatment effective at 6 or 12 months, four observed significant effects at shorter term follow-ups. During one study^45^ all participants received 12 weeks of nicotine patches and were assigned to one of four conditions: patch only, relapse prevention plus patch, CM plus patch and relapse prevention, or CM plus patch. At 12 weeks, participants assigned to receive CM showed statistically higher rates of PPA than those not assigned to receive contingencies.

Another intervention^30^ randomized outpatient smokers from a drug and alcohol dependence treatment program to counseling plus NRT or substance use treatment as usual. The PPA rates were significantly higher in the smoking cessation condition rather than treatment as usual, during week 2–7 but not at 6-month follow-up.

When comparing a brief 15-minute counseling session with three intensive 60-minute individual sessions and 8 weeks of nicotine patches, PPA was significantly higher for the intensive condition (27.5%) than the brief condition (6.6%) at 1 month postquit date but not at 6-month follow-up for outpatient alcohol dependent smokers.^49^

Finally, Patten and colleagues^43^ compared counseling plus nicotine gum, counseling plus physical exercise and a standard treatment condition for outpatient heavy smokers recovering from alcohol dependence. Short term differences in PPA rates between interventions were significant at posttreatment, with higher quit rates in the counseling plus physical exercise condition (60%) compared with standard condition (31%).

For the eight studies where no evidence of effectiveness was found, interventions involved counseling,^28^ NRT,^41^ CBT,^29^ motivational interviewing,^44^ bupropion,^39,42^ varenicline,^47^ and a combination of treatments.^46^ In relation to inpatient versus outpatient settings, one of the five inpatient studies found significant effects compared with eight of the 12 outpatient studies.

### Effects on Substance Use Outcomes

Ten of the 17 studies reported the impact of smoking cessation interventions on substance use outcomes. Two of these studies reported a difference in substance use outcomes across conditions. The drug and alcohol abstinence rates were higher in the MST condition rather than the MST+G condition at 6- and at 12-month follow-up,^33^ although neither differed significantly from that of the usual care condition (figures not reported in article). There was also a significant difference in drug and alcohol relapse rates between MST and MST+G conditions suggesting that participants in the MST condition had lower relapse rates than those in the MST+G condition. In the Shoptaw study described above,^45^ during weeks where patients with substance use disorders met the criteria for smoking abstinence they also provided more opiate and cocaine-free urine samples. The remaining eight studies did not find any significant difference between conditions in relation to substance use outcomes.

### Quality Review

Overall, many of the details required to determine quality were not reported (Table 2). Only eight studies reported sufficient information on randomization and three provided adequate information on allocation concealment. Nine studies reported adequate information on blinding of participants or investigators and seven reported adequate information on blinding of outcome assessors. Thirteen studies used intention to treat analysis^51^ where all randomized patients were included in their originally assigned groups and missing data was recorded as non-abstinence.

**Table 2.** Quality Review

	Selection bias		Performance bias	Detection bias	Attrition bias	Reporting bias	Sample bias
Author	Random sequence generation	Allocation concealment	Blinding of participant and personnel	Blinding of outcome assessment	Incomplete outcomes data	Selective reporting	Power analysis
Burling et al.^32 ^	Unclear risk	Unclear risk	Unclear risk	Unclear risk	Low risk	High risk	Not reported
Carmody et al.^38 ^	Low risk	Unclear risk	High risk	Unclear risk	Unclear risk	Low risk	Not reported
Cooney et al.^34 ^	Low risk	Low risk	Low risk	Low risk	Low risk	Low risk	Not reported
Cooney et al.^33 ^	Unclear risk	Unclear risk	Unclear risk	Unclear risk	High risk	High risk	>0.80
Gariti et al.^28 ^	Unclear risk	Unclear risk	Unclear risk	Low risk	Low risk	High risk	Not reported
Hays et al.^39 ^	Unclear risk	Unclear risk	Low risk	Unclear risk	Unclear risk	Low risk	Not reported
Hughes et al.^40 ^	Unclear risk	Unclear risk	Unclear risk	Unclear risk	Low risk	High risk	0.80
Kalman et al.^41 ^	Unclear risk	Unclear risk	Low risk	Low risk	Low risk	High risk	Not reported
Kalman et al.^42 ^	Low risk	Low risk	Low risk	High risk	Low risk	Low risk	0.80
Mueller et al.^29 ^	Unclear risk	Unclear risk	Unclear risk	Unclear risk	High risk	High risk	Not reported
Patten et al.^43 ^	Unclear risk	Unclear risk	Unclear risk	Unclear risk	Low risk	High risk	Not reported
Reid et al.^30 ^	Low risk	Unclear risk	Low risk	Unclear risk	Low risk	High risk	Not reported
Rohsenow et al.^44 ^	Low risk	Low risk	Low risk	Low risk	Low risk	Low risk	Not reported
Shoptaw et al.^45 ^	Low risk	Unclear risk	Unclear risk	Unclear risk	Low risk	High risk	Not reported
Stein et al.^46 ^	Unclear risk	Unclear risk	Unclear risk	Low risk	Low risk	High risk	0.80
Stein et al.^47 ^	Unclear risk	Unclear risk	Low risk	Low risk	Low risk	Low risk	0.80
Winhusen et al.^48 ^	Unclear risk	Unclear risk	Low risk	Unclear risk	Low risk	High risk	Not reported

Two studies had a high risk of reporting fewer outcomes than expected or not reporting the use of a protocol. Only five studies reported carrying out power analysis in sufficient detail within the article with all stating that they sought a power of at least .80. Effect sizes^36^ could only be calculated for 12 of the studies where the required information was reported. Of the studies with a significant effect, two studies^31,48^ had effect sizes between *r* = .17 and *r* = .19 and another had *r* = .27.^33^ Two studies had effect sizes^32,40^ between *r* = .32 and *r* = .47. Three studies without a significant effect at long term follow-up^30,43,46^ had effect sizes *r* < .1, three had effect sizes between *r* = .10 and *r* = .19.^28,47,49^

## Discussion

Seventeen randomized controlled trials investigating the effectiveness of smoking cessation interventions, for patients with substance use disorders were identified for inclusion in this review. Five studies reported significant effects on smoking cessation, providing evidence of effectiveness of NRT, behavioral support and combinations of the two, although not all trials testing combination treatments found an effect. Four other studies reported significant intervention effects at shorter follow-ups but not at the required 6 or 12 months, providing weaker evidence to support the effectiveness of NRT, combinations of NRT and behavioral support, as well as some suggestion that CM and physical activity may be beneficial. Two studies showed some evidence of improved substance use outcomes. They had in common that the smoking cessation interventions provided a combination of CM and relapse prevention. None of the trials suggested a negative effect of smoking cessation treatment on substance use outcomes.

Weaknesses in methods and reporting in some cases, combined with the small number of studies make the conclusion about the effectiveness of these interventions tentative. Many papers did not clearly state how the trials protected against bias, as indicated by the high proportion categorized as “unclear risk.”^52,53^ Only five studies reported carrying out a power analysis prospectively to determine sample size and it is particularly important to allow for attrition when treating patients with substance use disorders. When calculated, studies reporting significant differences had between small and medium effect sizes.^36^ Five of the nonsignificant results had very small effect sizes (<0.1) and would have required a much larger sample size (>700 participants) to achieve adequate power. Alternatively, a more intensive design or longer treatment may have produced stronger effects. None of the studies provided an economic evaluation of the interventions, which is an important factor in determining optimal treatment.

Meta-analysis could not be conducted due to the heterogeneity in the studies including the design, follow-up period and treatment lengths. Due to this we were not able to combine the effect sizes and probabilities found in the studies or combine results of studies to increase the power of statistical tests.^54^

Strengths of this review include that it concentrated on randomized controlled trials. This was due to the volume of study designs in this area and the aim of reviewing the most methodologically sound studies. Also, an established quality rating scale^35^ was used for data extraction and was completed independently by two authors to minimize any rating errors. The risk of publication bias was minimized through the use of several search mechanisms which strengthen the search strategy. The broad search strategy gives confidence that all currently available evidence has been identified in this review.

These findings update and extend previous review findings as 13 of the studies were conducted between 2003 and 2014 since the last meta-analysis was carried out.^11^ This review provides further descriptions of interventions and control conditions as well as recruitment methods, study staff, setting and treatment length to characteristics of a variety of treatment approaches.

NRT and behavioral support and combinations were effective in this population, which is in line with findings in the general population^17,18,55,56^ and the recommendations of the previous review for patients with substance use disorders.^11^ Varenicline was not found to be effective in one trial, which is different from the general population, where it has been found to be one of the most effective medications.^57^ We also found no evidence for the effectiveness of bupropion, which is effective in the general population,^58^ although again, there was only two trials investigating this in a substance use population.

Future research should include additional studies of the effectiveness of varenicline and bupropion for substance use disorder populations and address the methodological limitations of the studies included in this review. The aim should be to identify interventions that promote longer term abstinence into the 6- or 12-month follow-up period, using a measure of continuous abstinence wherever possible. Sample sizes should be planned prospectively to ensure adequate power to identify beneficial interventions. Future research should aim to identify which specific aspects of the combination treatments have a significant effect on smoking outcomes and what effect the mode of delivery or frequency of treatment have on abstinence in this population.

The timing of smoking cessation interventions is an important area of research as the question still remains whether smoking cessation treatment should be offered during substance use disorder treatment or delayed until treatment is complete.^24^ We did not test this hypothesis in our study but four of the effective studies offered concurrent smoking cessation and substance use disorder treatment suggesting that concurrent treatment of smoking alongside other treatment can be successful.

Barriers to implementing smoking cessation interventions for patients with substance use disorders include limited knowledge of how to engage this population into treatment and a belief that stopping smoking may affect recovery from other substances.^59^ Incorporating smoking cessation into substance use disorder treatment gives a clear message that quitting smoking is a major health priority. Patients with substance use disorders may require more intensive interventions to treat a number of addictions and tobacco is often viewed as a less harmful alternative to drugs and alcohol.^12^ Besides its addictive properties, cigarette smoking is legal and socially acceptable in many settings, giving it greater availability than other drugs.^60^ Treatment providers may need to employ strategies to avoid attrition, maintain motivation and make it as easy as possible to access the clinic. Modern smoking cessation approaches could be investigated; for example, e-cigarettes and mobile apps have yet to be tested as interventions in this population although risks and benefits associated with e-cigarette use are still under investigation.

## Conclusions

Smoking cessation interventions using NRT, behavioral support and combination approaches appear to increase smoking abstinence in those treated for substance use disorders and have no effect on other substance use treatment outcomes. However, higher quality studies and reporting are required to strengthen the evidence base.

## Supplementary Material

Supplementary Material S1 and Tables S2 and S3 can be found online at http://www.ntr.oxfordjournals.org

## Funding

UK Centre for Tobacco & Alcohol Studies, a UK Clinical Research Collaboration Public Health Research Centre of Excellence. Funding from the Medical Research Council, British Heart Foundation, Cancer Research UK, Economic and Social Research Council and the National Institute for Health Research under the auspices of the UK Clinical Research Collaboration (MR/K023195/1).

## Declaration of Interests

*None declared*.

## Supplementary Material

Supplementary Data
