# Supplementary material for: A Systematic Review of Smoking Cessation Interventions for Adults in Substance Abuse Treatment or Recovery
Source: Nicotine Tob Res. 2015 Jun 11;18(5):993–1001. doi: 10.1093/ntr/ntv127 (PMC4826485; doi:10.1093/ntr/ntv127)
Supplement: Supplementary Data [file supp_18_5_993__index.html]

A Systematic Review of Smoking Cessation Interventions for Adults in Substance Abuse Treatment or Recovery — A Systematic Review of Smoking Cessation Interventions for Adults in Substance Abuse Treatment or Recovery — A Systematic Review of Smoking Cessation Interventions for Adults in Substance Abuse Treatment or Recovery — A Systematic Review of Smoking Cessation Interventions for Adults in Substance Abuse Treatment or Recovery — Supplementary Data 

# A Systematic Review of Smoking Cessation Interventions for Adults in Substance Abuse Treatment or Recovery

## Supplementary Data

Data files

- Supplementary Data - Supplementary Data
- Supplementary Data - Supplementary Data
- Supplementary Data - Supplementary Data
