# Supplementary material for: A Systematic Review of Smoking Cessation Interventions for Adults in Substance Abuse Treatment or Recovery
Source: Nicotine Tob Res. 2015 Jun 11;18(5):993–1001. doi: 10.1093/ntr/ntv127 (PMC4826485; doi:10.1093/ntr/ntv127)
Supplement: Supplementary Data [file supp_ntv127_S2_excluded_references_070515.docx]

**Table S2: Excluded studies with reasons**

| **Reference** | | **Reason for exclusion** |
| --- | --- | --- |
| Alessi (2008) | Contingency Management promotes smoking reductions in residential substance abuse patients. | Not measuring the required Smoking outcome measure |
| Baltieri 2009 | Effects of topiramate or naltrexone on tobacco use among male alcohol dependent outpatients | Not designed to measure smoking behaviour and didn’t collect CO or nicotine withdrawal status |
| Berstein 2013 | Efficacy of an ED-Based Multicomponent Intervention for smokers with Substance Use Disorders | Only 3 Month follow-up |
| Bernstein 1999 | Adding a choice-based program for tobacco smoking to an abstinence-based addiction treatment program | No trial |
| Bobo 1996 | Predictors of tobacco quit attempts among recovering alcoholics | Only analysed whether quit attempt made or not. |
| Bobo 1996 | Nicotine dependence and intentions to quit smoking in three samples of male and female recovering alcoholics and problem drinkers | Survey |
| Bobo 1998 | Effect of smoking cessation counselling on recovery from alcoholism: findings from a randomized community trial | Not measuring the required Smoking outcome measure |
| Burling 1991 | Smoking cessation for substance abuse inpatients. | Only looking at stop smoking vs delayed rather than intervention type |
| Dunn 2010 | A contingency-management intervention to promote initial smoking cessation among opioid-maintained patients | Only 3 Month follow-up |
| Dunn 2008 | Voucher-based contingent reinforcement of smoking abstinence among methadone-maintained patients: a pilot study. | Only 3 Month follow-up |
| Dunn 2011 | Voucher-based contingent reinforcement of smoking abstinence among methadone-maintained patients: a pilot study | Recap of the findings from 2008 and 2010 papers |
| Carmody 2012 | Expectancies Regarding the Interaction Between Smoking and Substance Use in Alcohol-Dependent Smokers in Early Recovery | Results reported in the other 2012 paper |
| Easton 2007 | Age of onset of smoking among alcohol dependent men attending substance abuse treatment after a domestic violence arrest | No smoking cessation trial |
| Frosch 2002 | Optimizing smoking cessation outcomes among the methadone maintained | Shoptaw et al 2002 included as main paper |
| Friend 2005 | Smoking cessation and alcohol consumption in individuals in treatment for alcohol use disorders | No treatment for cigarette use |
| Fu 2008 | Ethnic differences in alcohol treatment outcomes and the effect of concurrent smoking cessation treatment | Concurrent or delayed treatment rather than two interventions |
| Grant 2003 | Smoking Cessation in Outpatient Alcohol Treatment | No biochemical verification |
| Grant 2007 | Bupropion and nicotine patch as smoking cessation aids in alcoholics | No trial |
| Grossman 2008 | 5 A's smoking cessation with recovering women in treatment | No trial |
| Hughes 1993 | Treatment of smoking cessation in smokers with past alcohol drug problems | Looking at positive history vs negative history rather than an intervention |
| Hurt 1994 | Nicotine dependence treatment during inpatient treatment for other addictions - A prospective intervention trial | Not randomised and compares alcoholics with non-alcoholics rather than treatment effectiveness |
| Indig 2013 | Heroin use impairs smoking cessation among Australian prisoners | Comparing prior heroin use to no use of heroin rather than a intervention |
| Joseph 1993 | Effect of treatment for nicotine dependence on alcohol and drug treatment outcomes. | No smoking assessment data |
| Joseph 1993 | Nicotine treatment at the Drug Dependency Program of the Minneapolis VA Medical Center. A researcher's perspective | Questionnaire and telephone interview and no trial |
| Hurt 1995 | Nicotine patch therapy for smoking cessation in recovering alcoholics | Recovering vs non alcoholics rather than an intervention |
| Joseph 2004 | A randomized trial of concurrent versus delayed smoking intervention for patients in alcohol dependence treatment | Concurrent or delayed treatment rather than two interventions |
| Kalman 2001 | Concurrent versus delayed smoking cessation treatment for persons in early alcohol recovery - A pilot study | Concurrent or delayed treatment rather than two interventions |
| Karam - Hage | Bupropion-SR for smoking cessation in early recovery from alcohol dependence: a placebo-controlled, double-blind pilot study. | Only 3 month follow-up |
| Khara 2010 | The Tobacco-Dependence Clinic: Intensive Tobacco-Dependence Treatment in an Addiction Services Outpatient Setting | No trial |
| Kohn 2003 | Changes in smoking status among substance abusers: baseline characteristics and abstinence from alcohol and drugs at 12-month follow-up | Not measuring the required Smoking outcome measure |
| Metrik 2011 | Marijuana use and tobacco smoking cessation among heavy alcohol drinkers | Looks at differences between current marijuana users |
| Nieva 2011 | Simultaneous versus Delayed Treatment of Tobacco Dependence in Alcohol-Dependent Outpatients | Concurrent or delayed treatment rather than two interventions |
| Okoli 2011 | Sex differences in smoking cessation outcomes of a tailored program for individuals with substance use disorders and mental illness | No trial |
| Richter 2005 | Dual pharmacotherapy and motivational interviewing for tobacco dependence among drug treatment patients | No trial |
| Reid 2011 | Smoking Cessation Treatment among Patients in Community-Based Substance Abuse Rehabilitation Programs: Exploring Predictors of Outcome as Clues Toward Treatment Improvement | Results reported in 2008 paper |
| Rohsenow 2002 | Brief interventions for smoking cessation in alcoholic smokers | This paper only reports the first 126 participants full results in the 2014 Rohsenow paper |
| Sonne 2010 | The Relationship between Depression and Smoking Cessation Outcomes in Treatment-Seeking Substance Abusers | Parent study included  Reid 2008 |
| Winhusen 2012 | Design considerations for a study to evaluate the impact of smoking cessation treatment on stimulant use outcomes in stimulant-dependent individuals | Winhusen 2013 reports the full findings of the trial |
| Shealy 2014 | Integrating smoking cessation into substance use disorder treatment for military veterans: Measurement and treatment engagement efforts | No trial |
| Tuten | Contingent incentives reduce cigarette smoking among pregnant, methadone-maintained women: results of an initial feasibility and efficacy randomized clinical trial. | No follow-up at 6 or 12 months |
| Winhusen 2014 | Achieving smoking abstinence is associated with decreased cocaine use in cocaine-dependent patients receiving smoking-cessation treatment | Results reported in 2013 paper |
| **Excluded from Reference check** | | |
| Schmitz 1995 | Contingent reinforcement for reduced carbon monoxide levels in methadone maintenance patients. | No trial |
| Shoptaw 1996 | Contingency management for tobacco smoking in methadone-maintained opiate addicts. Addictive Behavior. | No trial |
| Story 1991 | Treating cigarette smoking in methadone maintenance clients. | Not a smoking intervention they increased methadone dose |
| Margolin 1995 | A multicenter trial of bupropion for cocaine dependence in methadone-maintained patients. | Not smoking |
| Margolin 1990 | An open pilot study of bupropion and psychotherapy for the treatment of cocaine abuse in methadone-maintained patients | Not smoking |
| Hays 1999 | Response to nicotine dependence treatment in smokers with current and past alcohol problems | Alcoholics vs non alcoholics rather than effectiveness of smoking intervention |
| Hughes 2000 | Nicotine is more reinforcing in smokers with past alcoholism than in smokers without this history | Compares past history of alcoholism with no history of alcoholism not a smoking intervention |
| Schroeder 2009 | Confronting a Neglected Epidemic: Tobacco Cessation for Persons with Mental Illnesses and Substance Abuse Problems | No trial |
| Bobo 1995 | Nicotine addiction counseling for chemically dependent patients, | Professionals not patients and no trial |
| Patten 1998 | Effectiveness of cognitive-behavioral therapy for smokers with histories of alcohol dependence and depression | Mood management intervention with depression |
| Mutschler 2002 | An inpatient study of the effects of buprenorphine on cigarette smoking in men concurrently dependence on cocaine and opioids. | No trial |
| Hurt 2003 | Nicotine patch therapy based on smoking rate followed by bupropion for prevention of relapse to smoking | Oncology rather than substance abuse |
| Hurt 1996 | Mortality following inpatient addictions treatment. Role of tobacco use in a community- based cohort. | No trial |
| Hurt 2005 | Treating nondepressed smokers with alcohol dependence in sustained full remission: Nicotine patch therapy tailored to baseline serum cotinine | No trial |
| Olbrich 2008 | Smoking reduction during inpatient alcohol detoxification: a controlled clinical pilot | Not randomised |
| Poling 2006 | Six-month trial of bupropion with contingency management for cocaine dependence in a methadone-maintained population. | No smoking intervention |
