# Supplementary material for: A Systematic Review of Smoking Cessation Interventions for Adults in Substance Abuse Treatment or Recovery
Source: Nicotine Tob Res. 2015 Jun 11;18(5):993–1001. doi: 10.1093/ntr/ntv127 (PMC4826485; doi:10.1093/ntr/ntv127)
Supplement: Supplementary Data [file supp_ntv127_S3_Intervention_Characteristics.docx]

| **Intervention type** | **Author** | **Intervention (n)** | **Control (n)** | **Staff** | **Recruitment method** | **Substance Abuse Outcome** | **Smoking Cessation**  **Outcome^e1^** |
| --- | --- | --- | --- | --- | --- | --- | --- |
| **Counselling** | Cooney et al 200735 | Intensive smoking cessation treatment = x3, 1 hour individual counselling sessions, 8 weeks of nicotine patches 21 mg for 4 weeks then 14mg for 2 weeks then 7mg for 2 weeks (n = 55). | Brief smoking cessation advice = x1 15 minute counselling session with 5 minute follow-up.8 weeks of nicotine patches 21 mg for 4 weeks then 14mg for 2 weeks then 7mg for 2 weeks (n = 63). | Masters or doctoral level clinicians with experience in behavioral smoking cessation treatments. | Not reported | Proportion days heavy drinking  (f_( 2,95)_ = 0.19, p >.05, r =.04).  Prolonged Alcohol abstinence (χ^2^ _(1)_ = 0.06, p >.05, N = 95, r =.03). | ppa: at six month follow-up(χ^2^_(1)_ = 2.12, p >.05, N = 91, r =.15).  ppa : at 1 month (χ^2^ _(1)_ = 8.99, p<.01, N =112, r=.28) |
|  | Gariti et al 2002 29 | Structured motivational enhancement program for 16 weeks. One session with addiction therapist. Group film session on hazards of smoking and nicotine patches (n=34). | Usual care for 16 weeks including nicotine patches and referral to an outpatient cessation appointment (n=30). | Technician Trained therapists Research staff Study co-investigator nurse practitioner. | Not reported | Abstinence/  reduction of alcohol or drugs p>.05 (nr) | ppaat 6 month follow-up (χ^2^ _(1)_ = 0.002, p>05, N = 64, r = .12) |
| **Counselling and nicotine patches** | Reid et al 2008 31 | Smoking cessation (SC) group counselling x9 sessions as well as substance abuse TAU 21mg patches for 6 weeks, 14 mg for 2 weeks(n=153). | Substance abuse treatment as usual and offered deferred smoking cessation after completing their substance abuse treatment(n=72). | Counsellors | Word of mouth, use brochures and flyers, and referral from research clinic staff. | Abstinence from primary substance (f _(1, 1632)_ = 0.82, p>.05, r =.02). | ppa at week 26 (χ^2^ _(1)_ = .002, p>.05, N = 210, r = .003).  ppa during weeks 2-7:  *(f* (1, 1724) = 2.81 N = 225 p<.01 r = .04). |
| **Counselling and nicotine gum** | Patten et al 2001 44 | 12 weeks of treatment to either:  a) Behavioural counselling exercise (n=72).  b)Behavioural counselling plus nicotine gum (n=63). | 20 day quit programme, counselling and nicotine anonymous meetings (n=70). | Trained PHD and Masters level smoking health educators. | Flyers, word of mouth, AA and other 12-step meetings and clubs. | n/a | ppa at 12 months. (χ^2^ _(2)_ = .068, p>.05, N = 205, r =.02).  ppaat post treatment  (χ^2^_(2)_ = 17.85, p<.01, N = 205, r = .30) |
| **21 mg nicotine patch** | Hughes et al 200345 | 21 mg nicotine patch for 6 weeks, 14mg for 2 weeks, 7mg for 2 weeks and placebo for 2 weeks. 1 hour behavioural therapy group session (x6) then 15 minute individual sessions (x3)(n=61). | Placebo nicotine patches for 12 weeks and the same behavioural therapy (n=54). | Graduate students in psychology Trained tobacco cessation counsellors | Newspaper and radio advertisements, via flyers at outpatient alcohol treatment sites and at Alcoholics Anonymous meetings. | n/a | CAat4 months: (28% vs 11% OR 3.2 p<0.05, r=.27)  CA at 6 months:follow-up (24% vs 6%; OR 4.9, p<.05) r=.33. |
| **42 mg nicotine patch** | Kalman et al 2006 41 | 12 weeks 42 mg nicotine patches and x5 weekly 1 hour counselling sessions (n= Not reported). | 12 weeks 21 mg placebo nicotine patches and x5 weekly 1 hour counselling sessions (n= Not reported). | Pre-doctoral level Clinician | Not reported | n/a | ppaat any time point. 21 mg 19.9% 42 mg 9.2% p>.05 (nr). |
| **Intervention type** | **Author** | **Intervention (n)** | **Control (n)** | **Staff** | **Recruitment method** | **Substance Abuse Outcome** | **Smoking Cessation Outcome** |
| **2mg nicotine gum** | Cooney et al 200934 | 12 weeks 2mg nicotine gum and active nicotine patch x16 CBT sessions (n=45). | 12 weeks Placebo Gum, Active Nicotine patch, x16 CBT sessions(n= 51). | Therapists with a masters or doctoral degree and experience in cognitive behavioural addiction therapy. | Radio and newspaper advertisements and by referrals from the substance abuse clinic. | Continuous abstinence of alcohol, 90 days prior to follow up p>.05 (nr) | CA at 12 months:(χ^2^ _(1)_ = 7.25, p <.01, N = 96, r = .27). |
| **CBT, nicotine patches and lozenges** | Carmody et al  201239 | 16 sessions of Cognitive Behavioural Therapy and 16 weeks of nicotine patches and 26 weeks nicotine lozenges (n=82). | Referral to smoking cessation clinic (n=80). | Therapist | Not reported | 30 day alcohol abstinence rates p>.05 (nr) | ppaat 26 weeks: (p=.03)  Interaction effects of condition t^2^ _(139)_ = 2.30, p< .05, N = 119, r = .19, condition x time = t^2^ _(139)_ = 2.06, p< .05, N = 119, r = .17. |
| **CBT** | Mueller et al 201230 | Cognitive Behavioural Therapy x5 30 minute group sessions. 21 mg patches (only during inpatient treatment) (n=53). | Relaxation technique autogenic training 21 mg patches (only during inpatient treatment)  (n=50). | Psychologists trained in CBT with 3 years of experience in addiction treatment. | Not reported | Alcohol use p>.05 (nr) | ppa at 6 months:0% EG vs 6% CG p>.05 (nr) |
| **Motivational Interviewing** | Rohsenow et al 2014 49 | 45 minute initial motivational interview session followed by 2 booster sessions and NRT(n= 80). | 15 minute brief smoking advice session followed by 2 booster sessions and NRT(n=85). | Research Therapists. | Not reported | Number of days of alcohol or drug use p>.05 (nr) | ppaat 6 or 12 months : P>.05 (nr) |
| **Bupropion** | Hays et al 2009 43 | 52 weeks of treatment Bupropion SR 150 mg/day and brief behavioural counselling at each study visit (10 minutes) after 8 weeks of nicotine patches (n=56). | 52 weeks of placebo and brief behavioural counselling at each study visit (10 minutes) after 8 weeks of nicotine patches (n=54). | Research physician | News releases, advertisements Alcoholics Anonymous (AA) clubs and alcohol and drug treatment programs. Two members from the local AA community volunteered as study recruiters. | n/a | ppa at 12 months: p>.05 39.3% for bupropion vs 40.7% placebo.  CA 12 months: 41.1% for bupropion vs 40.7 for placebo p>.05 (nr) |
|  | Kalman et al 201142 | Bupropion SR 150mg,  Nicotine patches for 7 weeks and 8 weekly counselling sessions (n=73). | Placebo bupropion. Nicotine patches for 7 weeks and 8 weekly counselling sessions  (n=70). | First author provided counselling. | Not reported | n/a | ppa at 6 months:  Bupropion 6% placebo 11% p>.05.  CA at 6 months: bupropion 3 % placebo 5% p>.05 (nr) |
| **Intervention type** | **Author** | **Intervention (n)** | **Control (n)** | **Staff** | **Recruitment method** | **Substance Abuse Outcome** | **Smoking Cessation Outcome** |
| **Varenicline** | Stein et al 201350 | 24 weeks of treatment to either: a)Varenicline and standardised advice to quit. (n=137) b) Combination nicotine patches, nicotine gum and standardised advice to quit (n=133). | Varenicline –placebo and standardised advice to quit (n=45). | Research Staff | Not reported | n/a | ppa at 6 months:  (χ^2^ _(1)_ = 3.56, p >.05, N = 315, r = .11). |
| **Counselling, contingency management and relapse prevention.** | Burling et al 2001 33 | 9 weeks of treatment and daily counselling to either:  a)Multicomponent smoking treatment (MST) including multiple approaches (n= 50) b) Generalization training of cessation and relapse prevention skills to drug and alcohol use (MST+G)(n=50). | Usual care (UC) including access to nicotine patches or gum (N= 50). | Masters or Doctorate level Counsellors. | Not reported | Continuous drug and alcohol abstinence at six(χ^2^ _(1)_ = 4.91, p <.05, N =93, r =.23) and 12 months(χ^2^ _(1)_ = 4.27, p <.05, N=85, r =.22)  Significant difference in relapse rates (χ^2^ _(1)_ = 6.00, p <.05, N =100, r =.24) | ppa at6 and 12 months p>.05 (nr).  CA at 6 and 12 months. MST/MST+G vs UC χ^2^ _(1)_ = 19.07, p <.0001, N = 150, r = .36, MST vs UC = (χ^2^ _(1)_ = 22.22, p <.0001, N = 100, r = .47, MST+G vs UC χ^2^ _(1)_ = 13.79, p <.001, N = 100, r = .37). |
| **Contingency management, relapse prevention and nicotine patches** | Shoptaw et al 200246 | 12 weeks of treatment to either: a) Patch plus relapse prevention. (n=42)  b) Patch plus contingency management (n=43).  c) Patch plus relapse prevention and contingency management (n=47). | Patch only (n=43). | Counsellor | Flyers and counsellor nominations. | Urine sample to test for opiates (*f* (1, 2054) =14.38, N= 175, p=.0002, r =.08) and cocaine (*f* (1, 2419 =16.52, N=175, p<.0001, r = .08) significant | ppa at 6 or 12 months p>.05 (nr).  ppa at 12 weeks *f* (3,4680) = 6.3 N= 175 p<.05 r = .04 |
| **Bupropion, nicotine inhaler, counselling and contingency management** | Winhusen et al 201347 | 10 weeks of mixed method smoking cessation treatment including bupropion, nicotine inhaler, counselling sessions and contingency management (n=267). | Substance abuse treatment as usual. Weekly 10 minute counselling sessions (n=271) | Interventionists trained and certified on the manual. | Not reported | Proportion of Stimulant-abstinent participants (χ^2^ _(1)_ = 0.65, p>.05, N = 538, r = .03)  stimulant abstinence at 6 months (χ^2^ _(1)_ = 0.10, p>.05, N = 538,r = .01)  Stimulant free days at 6 months (χ^2^ _(1)_ = 1.26, p>.05, N = 538,r = .05) | ppaat 10 weeks: (χ^2^ _(1)_ = 44.69, p <.0001, N = 538,r = .29). 3 months (χ^2^ _(1)_ = 26.73, p <.0001, N = 479, r = .24)6 months 13% vs 4% (χ^2^ _(1)_ = 13.00, p=.0003, N = 428, r = .17). |
| **Brief motivational intervention, behavioral counselling, relapse prevention and nicotine patches** | Stein et al 200652 | Received up to 3 visits including a brief motivational intervention, setting a quit date and follow-up session including relapse prevention. Nicotine patches (n=191). | Up to two individual brief advice visits with a study interventionist using 4 A’s model. Nicotine patches (n=192). | PhD-level study interventionists. | Not reported | n/a | ppa at 6 months:5.2% vs 4.7 % (χ^2^ _(1)_ = .06, p>.05, N = 54, r = .03). |

^1^Primary outcome 6 or 12-month continuous abstinence from smoking

Secondary outcomes: 6 or 12 months point prevalence abstinence; shorter-term abstinence

(nr) = Figures not reported within the paper
