# Supplementary material for: A Systematic Review of Smoking Cessation Interventions for Adults in Substance Abuse Treatment or Recovery
Source: Nicotine Tob Res. 2015 Jun 11;18(5):993–1001. doi: 10.1093/ntr/ntv127 (PMC4826485; doi:10.1093/ntr/ntv127)
Supplement: Supplementary Data [file supp_ntv127_S1_Search_terms_070515.docx]

**S1: Search Terms**

**Ebschohost: CINAHL, Medline, Psycharticles, Psychbooks and Psychinfo**

“Smoking cessation” or “smoking” or “tobacco smoking” or “nicotine” or “tobacco use disorder” or “smoking cessation programme”

and

“Drug abuse” or “substance abuse” or “substance related disorder” or “substance abuse intravenous” or “alcohol abuse” or “drug rehabilitation” or “drug usage” or “drug dependence” or “drug addiction” or “substance use disorder” or “alcohol drinking” or “addiction” or “substance abuse treatment” or “alcohol rehabilitation” or “opioid related disorder”

**Dare, Swetwise, British Library:**“Substance abuse” and “smoking cessation”

**Cochrane Library: “**Substance abuse” and “smoking cessation treatment”

**Web of Science:**“Substance abuse” and “smoking cessation” (title and cited by)

**Science Direct:**“Smoking cessation” or “smok*” or “cigarette smoking” or “nicotine dependence” or “smoking abstinence” or “tobacco treatment” or “cessation effort” or “cessation treatment” or “quit smoking” or “smoking” or “tobacco dependence”.

And

“Substance abuse” or “abuse treatment” or “addiction disorder” or “alcohol counselling” or “drug abuse” or “drug treatment” or “substance abuser” or “substance abusing”

**Grey literature: OpenGrey, UK Medical Research Council, The Universal Index of Doctoral Dissertation, Ethos (British Library), Conference Proceedings Citation Index and Medical Research Council Grants and Fellowships awarded.**

“Substance abuse” and “smoking cessation”
